# Detection and prevalence of monoclonal gammopathy of undetermined significance: a study utilizing mass spectrometry-based monoclonal immunoglobulin rapid accurate mass measurement

**DOI:** 10.1038/s41408-019-0263-z

**Published:** 2019-12-13

**Authors:** David Murray, Shaji K. Kumar, Robert A. Kyle, Angela Dispenzieri, Surendra Dasari, Dirk R. Larson, Celine Vachon, James R. Cerhan, S. Vincent Rajkumar

**Affiliations:** 1 Department of Laboratory Medicine and Pathology, Mayo Clinic, Rochester, MN 55906 USA; 2 Division of Hematology, Mayo Clinic, Rochester, MN 55906 USA; 3 Department of Health Sciences Research, Mayo Clinic, Rochester, MN 55906 USA; 4 Division of Biostatistics, Mayo Clinic, Rochester, MN 55906 USA

**Keywords:** Cancer epidemiology, Lymphoproliferative disorders

## Abstract

High-sensitivity mass spectrometry assays are available to detect monoclonal immunoglobulins. To better assess the prevalence of monoclonal gammopathy of undetermined significance (MGUS), we identified 300 patients diagnosed with MGUS or related gammopathy who had a prior negative work-up for monoclonal proteins as part of the Olmsted County MGUS screening study. Two mass spectrometry-based detection methods (matrix-assisted laser desorption/ionization-time of flight (MALDI-TOF) and monoclonal immunoglobulin rapid accurate mass measurements (miRAMM) along with traditional immunofixation were performed on the Olmsted baseline and MGUS diagnostics serum samples. Among the 226 patients considered negative for MGUS based on protein electrophoresis and serum-free light-chain assay, a monoclonal protein could be detected at baseline in 24 patients (10.6%) by immunofixation, 113 patients (50%) by MADLI-TOF mass spectrometry, and 149 patients (65.9%) by miRAMM mass spectrometry. In addition, using miRAMM, some patients demonstrated an oligoclonal to monoclonal transition giving insight into the origin of MGUS. Using the sensitive miRAMM, MGUS is present in 887 of 17,367 persons from the Olmsted County cohort, translating into a prevalence of 5.1% among persons 50 years of age and older. This represents the most accurate prevalence estimate of MGUS thus far.

## Introduction

Monoclonal gammopathy of undetermined significance (MGUS) is a premalignant plasma cell disorder that is present in ~3–4% of the general population over the age of 50^1–3^. It is associated with a risk of progression to multiple myeloma (MM) or related disorder at a rate of 1% per year^4,5^. The prevalence estimates for MGUS have been based on testing using serum protein electrophoresis and subsequent confirmation of any abnormality detected on electrophoresis using serum immunofixation^1,3,6^. More recently, the prevalence of MGUS has also been refined using the serum-free light-chain (FLC) assay to detect light-chain MGUS^2^. Previous mathematical estimates suggest that when MGUS is first clinically recognized, it has likely been present in an undetected state for a median duration of >10 years^7^. To verify these estimates, a serum-based method with higher analytical sensitivity than SPEP is needed. Lower levels of monoclonal proteins (M-proteins) can be detected using mass spectrometry assays^8,9^. In addition to high-analytical sensitivity, mass spectrometric assays also enable accurate follow-up of the identified M-protein as the molecular weight of the M-protein light-chain is a specific and reliable marker of the plasma cell clone. We studied a cohort of patients who were part of the Olmsted County screening study to address this question. We hypothesized that a monoclonal protein can be detected with sensitive mass spectrometry assays in most patients several years prior to a diagnosis of clinical MGUS.

## Methods

### Study subjects

Details of the Olmsted County screening study have been previously published^1^. The original study cohort comprises samples from 21,463 of the 28,038 enumerated Olmsted County residents aged 50 or over as of 1 January, 1995. Of these, 17,367 patients comprised the identifiable Olmsted County screening cohort in whom testing for monoclonal protein was performed between 1 January, 1995, and 31 December, 2001. The testing consisted of serum protein electrophoresis on all samples. Any sample that had a definite or questionable abnormality was subjected to serum protein immunofixation (IFE) for definitive diagnosis of a monoclonal protein. MGUS (IgM or non-IgM) was identified in 605 of the 17,367 persons (3.5%). Subsequently, serum-free light-chain (FLC) assay was performed on all available samples, and these studies identified light-chain MGUS in an additional 133 persons (0.8%)^2^. Thus, the combined prevalence of MGUS (IgM, non-IgM, and light-chain types) was 4.24% (738 of 17,367 persons).

For this study, we queried the Mayo Clinic dysproteinemia database to identify patients who had no evidence of MGUS or light-chain MGUS as part of the screening study but were subsequently clinically diagnosed with MGUS or related monoclonal gammopathy over the next several years up to 30 June, 2014. This study was approved by the Mayo Clinic IRB. Clinical diagnosis of MGUS was based on positive serum IFE. We performed serum IFE on all patients diagnosed with clinical MGUS using baseline samples obtained at the time of the screening study to enable comparison of sensitivity to the mass spectrometry assays described below. A second cohort of patients who had no evidence of MGUS or light-chain MGUS as part of the screening study who were also negative a second time at least 1 year from the original study were identified. Mass spectrometry was performed on the original sample as a “double-negative” control.

### Mass spectrometry assays

#### Matrix-assisted laser desorption/ionization-time of flight (MALDI-TOF)

The MALDI-TOF mass spectrometry is currently used for clinical purposes at Mayo Clinic instead of conventional serum IFE for detection and isotyping of monoclonal proteins, and is referred to as “MASS-FIX”^8,10^. The methods for MALDI-TOF have been described in detail elsewhere. Briefly, the assay uses isotype-specific nanobody (NB) enrichment coupled to MALDI-TOF mass spectrometry. In addition to detecting and isotyping monoclonal proteins, the assay also enables accurate quantification of monoclonal protein, in effect providing the combined benefit of serum protein electrophoresis and immunofixation in one test^8^.

#### Monoclonal immunoglobulin rapid accurate mass measurement (miRAAM)

Electrospray‐ionization time‐of‐flight mass spectrometry (microLC‐ESI‐Q‐TOF MS) referred to as miRAMM is a highly sensitive method for the detection of monoclonal proteins in the serum and urine^9,11^. We performed the miRAAM assay on baseline serum samples interpreted as negative for monoclonal protein in the initial screening study (baseline serum), as well as on samples obtained at the time of clinical monoclonal gammopathy diagnosis (diagnostic serum). The assay methodology has been previously published^9^. Briefly, two serum immunoglobulin (Ig) enrichments were performed using a camelid-derived nanobodies targeting the constant domains of the heavy (i.e., IgA, IgG, or IgM) and light chains kappa and lambda (Thermo Fisher Scientific PN: 084910 and 083310), Life Technologies, Carlsbad, CA USA. After enrichment, samples were eluted with 20 μl of 5% acetic acid containing 50 mM tris [2-carboxyethyl] phosphine, to disassociate immunoglobulins into separated light-chain and heavy-chain components. An Eksigent Ekspert 200 micro liquid chromatography (Foster City, CA, USA) was used to separate immunoglobulin light chains before ionization and detection. AB SCIEX TripleTOF 5600 quadrupole TOF mass spectrometry using electrospray ionization in positive ion mode was used for miRAMM analysis. Data analysis was performed using Analyst TF v1.6 and PeakView ver. 2.2 (AbSciex, Framingham, MA, USA). The mass spectra of the multiply charged light-chain ions were deconvoluted to accurate molecular mass using the Bio Tool Kit ver. 2.2 plug-in software (AB Sciex, Framingham, MA, USA). The retention time of the monoclonal light chain in each sample was tracked using PeakView. Mass measurement accuracy was estimated to be 15 p.p.m. over the course of the analysis.

### Detecting M-proteins in spectra

In the mass spectra, the diagnostic serum was first inspected visually for a peak(s) consistent with the isotype detect by serum immunofixation. For example, if serum immunofixation determined an IgG kappa M-protein, a peak was expected for both the IgG enriched and kappa enriched diagnostic serum. For miRAMM, the peak masses all matched within +/−1 Da and +/−10 Da for MASS-FIX consistent with previous studies. All baseline serum were visually inspected for a peak(s) matching the mass of the peak from the diagnostic sample within +/−1 Da for miRAMM and +/−10 Da for Mass-Fix. If a peak was present in the baseline serum matching the mass of the diagnostic serum peak, the baseline serum was deemed positive.

To provide a more quantitative measure of our visual inspection, a method-specific signal to noise (*S*/*N*) was calculated on all miRAMM-positive samples. The *S*/*N* was calculated dividing the total area under the curve (AUC) in the deconvoluted light-chain mass range by AUC under the M-protein light-chain peak of interest. This corresponds to the percent AUC of the peak of interest in comparison to the total “polyclonal” AUC. *S*/*N* metric of 4.0 was established as lower limit for detecting an M-protein. The average *S*/*N* of an M-protein in baseline and diagnostic samples were 12.05 and 23.68, respectively. This is in contrast to the baseline of 50 patients in the double-negative cohort who did not demonstrate any peaks with a *S*/*N* > 2. Supplemental Fig. 1 illustrates the computation of *S*/*N* metric with examples.

### Statistical methods

We determined the proportion of patients who developed clinical MGUS or related monoclonal gammopathy in whom the origin of the monoclonal protein could be detected years prior to the diagnosis through the sensitive miRAMM assay. The study was designed, the data were gathered and analyzed, and the manuscript was written by all the authors. This study was conducted with the approval of the Mayo Clinic Institutional Review Board.

## Results

### Patient cohort

Of 16,629 identifiable patients in the Olmsted County Screening cohort who were negative for MGUS or light-chain MGUS during the initial screening period, monoclonal gammopathy was clinically diagnosed during subsequent follow-up in 300 patients. Of these, 226 (109 women, 117 men) had cryopreserved serum samples from the initial screening available for testing with mass spectrometry assays, and represent the study cohort. The median age at clinical diagnosis was 78.4 years (range, 54.4–96.3 years). The median time from initial negative screening result to the first clinical diagnosis of monoclonal gammopathy was 10.1 years (range 0.3–18.5 years). The first clinical diagnosis of monoclonal gammopathy was MGUS in 220 patients, and multiple myeloma in six patients. Since MGUS always precedes multiple myeloma, these patients were included as MGUS for the purposes of calculating revised prevalence estimates.

### Detection of monoclonal gammopathy by mass spectrometry in baseline samples

**Fig. 1:**
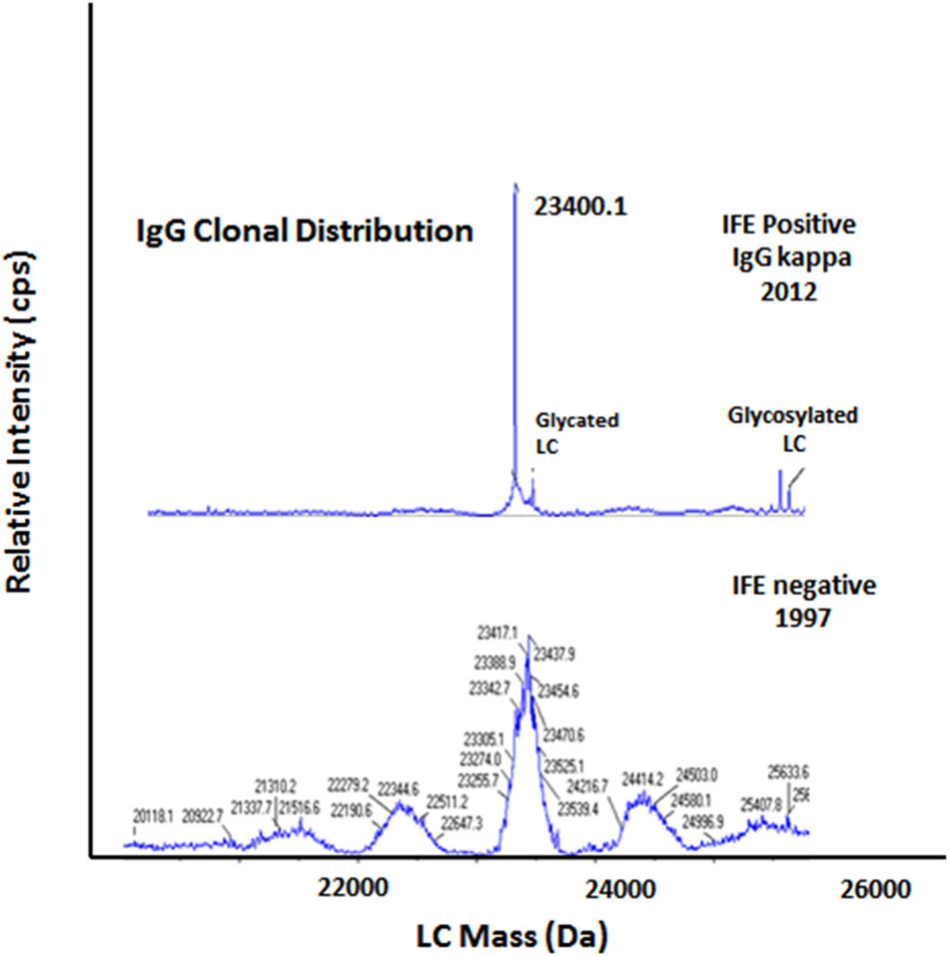
miRAMM findings in a patient who was considered negative at the time of screening in 1997 but developed a monoclonal protein in 2012. Top panel demonstrates an IgG kappa M-protein (23,400.1 Da) by miRAMM, also showing post-translational light-chain modification by glycation and glycosylation. Bottom panel in the same patient 15 years earlier, showing no evidence of monoclonal protein.

**Fig. 2:**
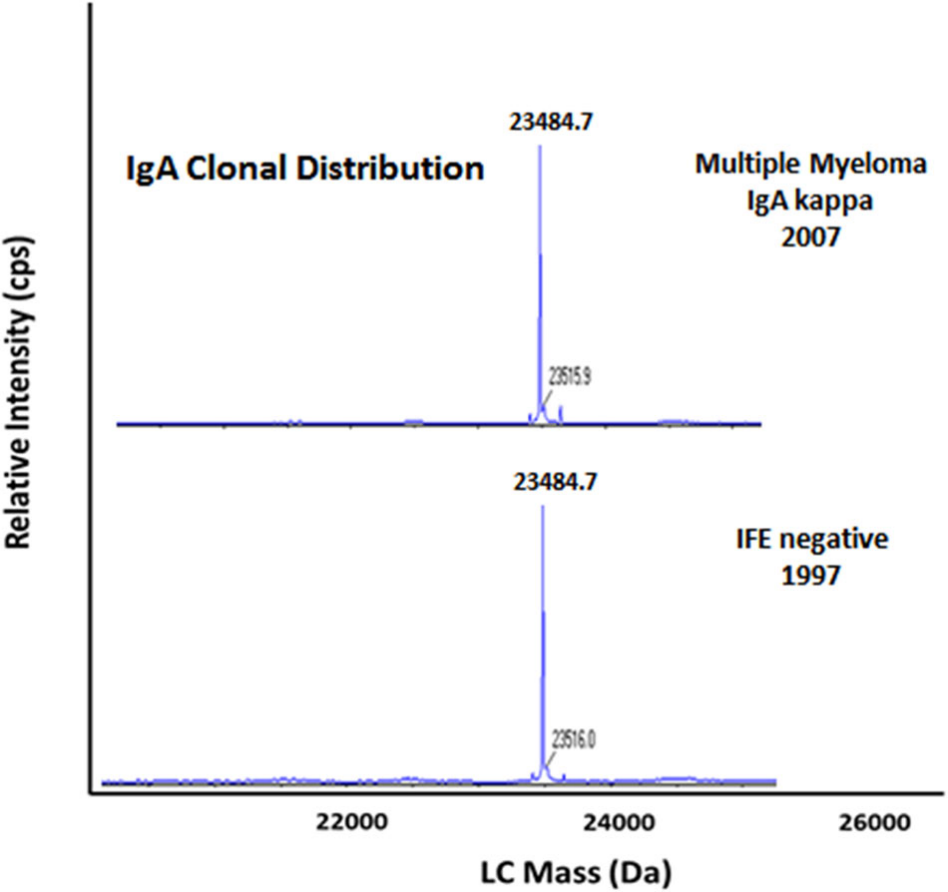
miRAMM findings in a patient who was considered negative at the time of screening in 1997 and detected to have a monoclonal protein by serum immunofixation in 2007. Top panel demonstrates an IgA kappa M-protein (23,484.7 Da) by miRAMM. Bottom panel in the same patient 10 years earlier, shows that the same monoclonal protein was detectable in 1997 by miRAMM.

We tested baseline samples from the time of the initial screening study with the MALDI-TOF and miRAMM mass spectrometry assays to determine the proportion of patients in whom a detectable monoclonal protein was present at baseline, but missed using our initial strategy of serum protein electrophoresis and serum FLC assay. Among the 226 patients who were considered negative for MGUS based on protein electrophoresis and serum-free light-chain assay, a monoclonal protein could be detected by in the baseline sample in 24 patients (10.6%) by IFE, 113 patients (50%) by MADLI-TOF mass spectrometry, and 149 patients (65.9%) by the miRAMM assay (Table 1). Figures 1 and 2 show the pattern of results observed with miRAMM mass spectrometry, illustrating likely true-negative at baseline (Fig. 1), and false-negative at baseline (Fig. 2).

**Table 1:** Detection of monoclonal protein using mass spectrometry assays among patients considered not to have monoclonal gammopathy of undetermined significance (MGUS) in the Olmsted Screening Study, but subsequently developed the disorder during follow-up.

	Assay type		
	Serum immunofixation	MALDI-TOF	miRAMM
Baseline samples considered negative for MGUS by serum protein electrophoresis and serum-free light-chain assay			
Number of patients tested	226	226	226
Number of patients with detectable monoclonal protein, (%)	24 (10.6)	113 (50.0)	149 (65.9)
Samples from time of clinical MGUS diagnosis			
Number of patients tested	226	226	221
Number of patients with detectable monoclonal protein, (%)	226 (100)^a^	188 (83.2)	200 (90.5)

*MGUS* monoclonal gammopathy of undetermined significance, *MALDI-TOF* matrix-assisted laser desorption/ionization-time of flight, *miRAMM* monoclonal immunoglobulin rapid accurate mass measurement

^a^By definition, clinical MGUS was diagnosed based on a positive serum immunofixation

### Detection of monoclonal gammopathy by mass spectrometry at time of clinical diagnosis of MGUS

**Fig. 3:**
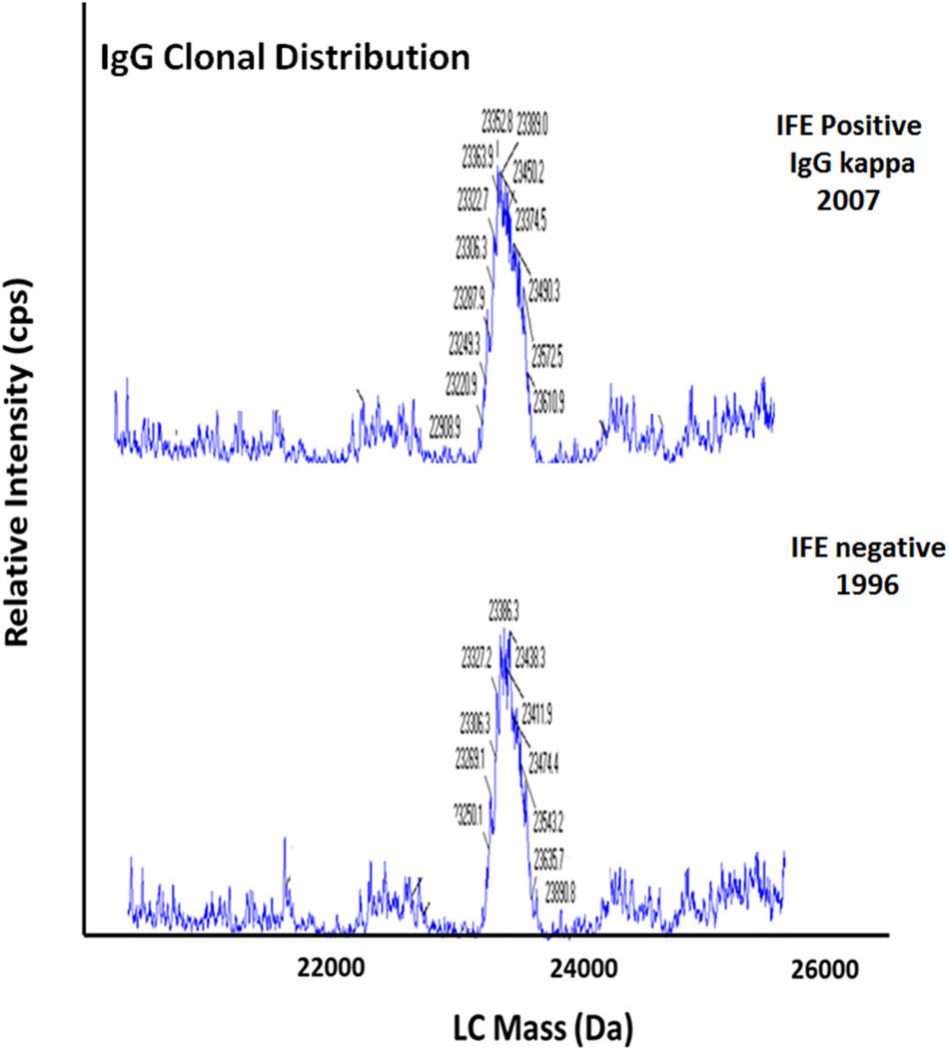
miRAMM showing no evidence of monoclonal protein in a patient considered to have positive immunofixation. Top panel demonstrates no monoclonal protein by miRAMM in 2007 in a patient considered to have IgG kappa monoclonal protein on immunofixation. Bottom panel in the same patient from 1996 shows no monoclonal protein and a pattern identical to the one seen in 2007.

The diagnosis of clinical MGUS was based on serum immunofixation. Using MALDI-TOF mass spectrometry, a monoclonal protein at the time of clinical MGUS was detected in 188 of 226 patients (83.2%). Serum samples were available in 221 patients from the time of clinical diagnosis for the miRAMM assay. The presence of a monoclonal protein was confirmed on the miRAMM assay in 200 (90.5%) patients. In the remaining 21 patients (9.5%), there was no detectable monoclonal protein on the miRAMM assay. None of these 21 patients had multiple myeloma. In 17 of the 21 patients a monoclonal protein was not detectable by serum protein electrophoresis or MALDI-TOF, but only identified on serum immunofixation, and hence may represent false-positives on immunofixation. Figure 3 illustrates miRAMM results, suggesting likely false-positive clinical diagnosis by immunofixation. In three patients, a monoclonal protein was detected by MALDI-TOF and serum immunofixation, but samples were insufficient for a conclusive analysis by the miRAMM assay. In the one remaining patient the monoclonal protein was detected on electrophoresis (monoclonal protein level 0.5 gm/dl) and immunofixation but was not detected by either the MALDI-TOF or miRAMM mass spectrometry assays. The method-specific signal to noise (*S*/*N*) statistics of the positive diagnostic and baseline sera were calculated, and demonstrated that all visually positive baseline samples have *S*/*N* levels above 2, the traditional *S*/*N* level used to define limit of detection^12^.

**Fig. 4:**
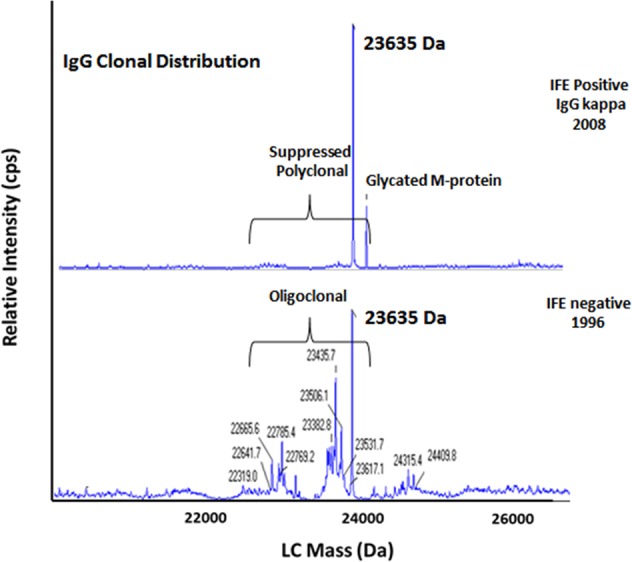
miRAMM showing origin of monoclonal gammopathy from within an oligoclonal response. Top panel shows an IgG kappa (23,635 Da) monoclonal protein with non-enzymatic glycation. Bottom panel shows same patient 12 years earlier demonstrating an oligoclonal profile with a clone matching the future MGUS clone (23,635 Da) detectable within the oligoclonal background.

**Fig. 5:**
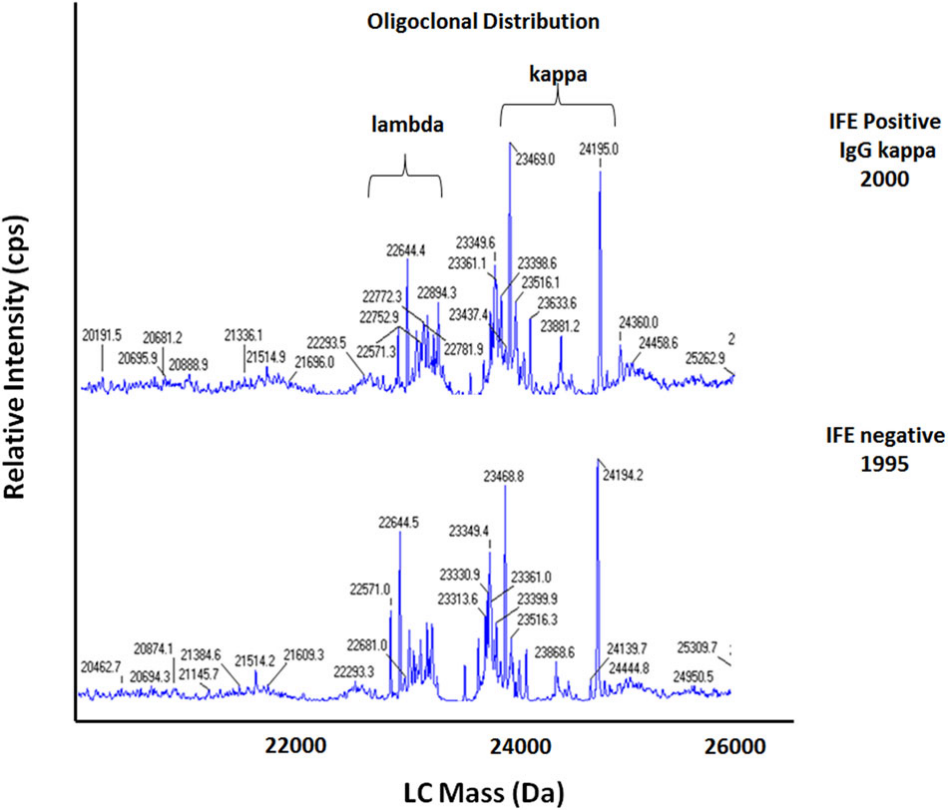
miRAMM showing persistence of an oligoclonal immune response over a 5-year period. Top panel: oligoclonal pattern in 2000, considered positive for IgG kappa monoclonal protein by immunofixation. Bottom panel: sample from 5 years in 1995 showing same oligoclonal distribution, with immunofixation considered negative during this period.

Figures 4 and 5 demonstrate the potential of miRAMM mass spectrometry to decipher the origins of MGUS, and possible role as a predictor for progression. In Fig. 4, MGUS appears to arise from an oligoclonal response, while Fig. 5 illustrates what is considered a monoclonal protein on immunofixation may be more of an oligoclonal response when visualized using miRAMM mass spectrometry.

### Prevalence of MGUS

Since we performed serum immunofixation and miRAMM assay on all baseline samples, the study allows us to calculate revised estimates for the prevalence of MGUS in the general population over the age of 50 years based on these more sensitive methods. If serum immunofixation is used as a screening strategy, 25 additional persons with MGUS would have been identified, increasing the prevalence of MGUS to 4.4% (763 of 17,367 persons). Using the miRAMM assay, 149 additional persons with MGUS would have been identified, translating into a prevalence of 5.1% (887 of 17,367 persons), which represents the most accurate prevalence estimate of MGUS thus far (Table 2).

**Table 2:** Estimated prevalence of monoclonal gammopathy of undetermined significance (MGUS).

Method	Estimated prevalence
Serum protein electrophoresis, and confirmation by immunofixation if any abnormality detected	3.5%
Serum protein electrophoresis plus serum-free light-chain assay	4.2%
Serum immunofixation plus serum-free light-chain assay	4.4%
miRAMM plus serum-free light-chain assay	5.1%^a^

*miRAMM* monoclonal immunoglobulin rapid accurate mass measurement

^a^This estimate represents the lower limit of the estimated prevalence of MGUS

## Discussion

The primary purpose of this study was to explore the epidemiology of MGUS using a more sensitive method. We found that a monoclonal protein can be detected by mass spectrometry (MALDI-TOF or miRAMM) in most patients several years prior to the diagnosis of clinical MGUS. MALDI-TOF was able to identify a monoclonal protein in 50% of patients who were considered negative by conventional methods, while miRAMM detected a monoclonal protein in approximately two-third of patients. These results confirm our previous mathematical estimates that when MGUS is first clinically recognized, it has likely been present in an undetected state for a median duration of >10 years^7^. Our study also shows that ~1% of patients with negative serum protein electrophoresis and serum-free light-chain measurements can develop clinically recognizable MGUS over a median duration of 10 years, giving some insight into the incidence of MGUS in an older, predominantly white patient population. More importantly, our study provides the first estimate of the prevalence of MGUS in the general population when the most sensitive methods for detecting monoclonal proteins are used. Based on our study, the prevalence of MGUS in persons over the age of 50 is at least 5.1% or higher. We recognize that the prevalence estimate would be higher if miRAMM was done on all patients at baseline, but this was not possible due to resource and sample constraints.

One of the main concerns in regards to the miRAMM method was that we would find MGUS in the majority of elderly population. This was not the case as the prevalence of MGUS was about 5% reassuring that the mass spec assay is affording the expected lead-time bias of a more sensitive assay. Currently, there is not a recommendation for screening the general population and the data from this study does not change this recommendation.

Our study also demonstrates the sensitivity of mass spectrometric methods in detection of monoclonal proteins. Since we included only patients who were detected to have clinical MGUS during follow-up, the results of baseline testing can be considered accurate since any abnormality on mass spectrometry can be verified with the gold standard molecular weight of the M-protein light-chain at the time of clinical diagnosis. Thus, we found that miRAMM is more sensitive for detection of monoclonal proteins than the MALDI-TOF mass spectrometry method. We have made similar observations in the past, and are hence developing the miRAMM assay as the preferred mass spectrometric method for minimal residual disease detection^13^.

Correlation with results at the time of clinical diagnosis shows that mass spectrometry, especially miRAMM, may be more specific than the current standard of immunofixation. We found that in 21 patients who were considered to have a monoclonal protein, none had myeloma, and most were negative for monoclonal protein by serum protein electrophoresis, serum-free light-chain assay, and by MALDI-TOF, and this is best illustrated in Fig. 3.

Our study provides interesting new data on the origin of MGUS. Since we studied a population who converted from “negative” to “positive”, we were able to see the initial MGUS clone arise in the setting of an oligoclonal immune response, which over time results in the establishment of a dominant MGUS clone (Fig. 4). We also hypothesize that the study of mass spectrometry patterns can distinguish true monoclonal proteins from those that appear monoclonal on immunofixation, but in reality are oligoclonal and hence may have a lower likelihood of disease progression.

The MALDI-TOF (Mass-Fix) method is currently being used instead of immunofixation electrophoresis in our clinical laboratory. Our first year of experience with this assay has confirmed that Mass-Fix is easy to interpret, suitable for M-protein quantitation, cost competitive to gel electrophoresis, and has increased the overall efficiency of our clinical lab staff. Efforts are also underway to commercialize the assay in order to make it available to other clinical labs. The miRAMM method, however, is very sensitive but is more labor and resource intensive in comparison to MALDI-TOF, and is probably best used for detection of minimal residual disease, and is currently only a research test.

## Supplementary information

Suppl Figure 1

Suppl Figure legend
