# Supplementary figures and images for: Detection and prevalence of monoclonal gammopathy of undetermined significance: a study utilizing mass spectrometry-based monoclonal immunoglobulin rapid accurate mass measurement
Source: Blood Cancer J. 2019 Dec 13;9(12):102. doi: 10.1038/s41408-019-0263-z (PMC6910906; doi:10.1038/s41408-019-0263-z)

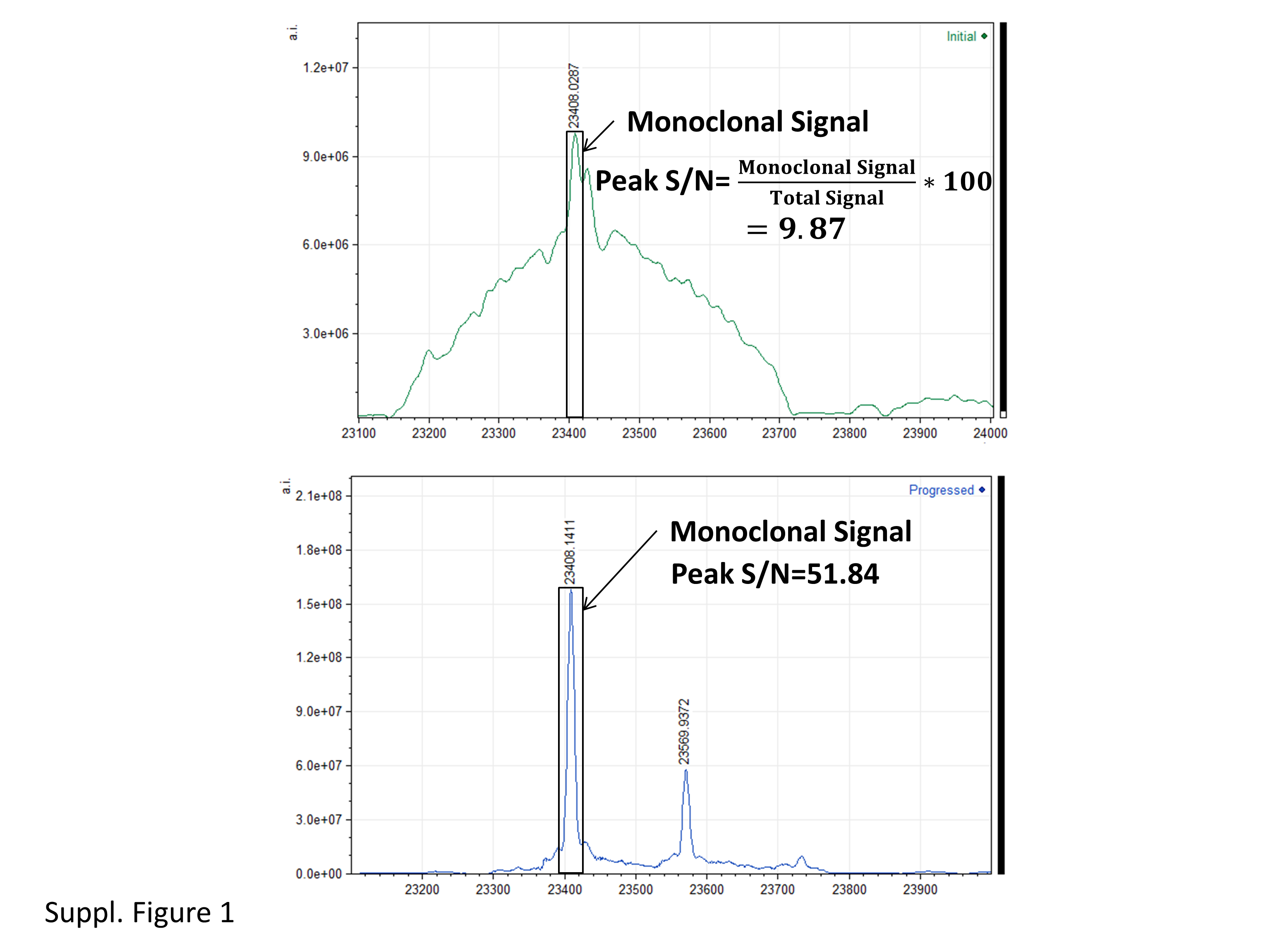

Supplement: Supplementary file 1 — Suppl Figure 1 [file 41408_2019_263_MOESM1_ESM.tif]
