# Supplementary material for: Detection and prevalence of monoclonal gammopathy of undetermined significance: a study utilizing mass spectrometry-based monoclonal immunoglobulin rapid accurate mass measurement
Source: Blood Cancer J. 2019 Dec 13;9(12):102. doi: 10.1038/s41408-019-0263-z (PMC6910906; doi:10.1038/s41408-019-0263-z)
Supplement: Supplementary file 2 — Suppl Figure legend [file 41408_2019_263_MOESM2_ESM.docx]

Suppl. Figure 1

Example of the calculation of signal to noise (s/N) on a study participant who was negative by IFE but had a low level monoclonal protein on initial screen by miRAMM (top) and who subsequently became IFE positive (lower). The same S/N calculation was performed on all miRAMM data performed on the entire cohort.
